# Validation of the German version of the needs assessment tool: progressive disease-heart failure

**DOI:** 10.1186/s12955-021-01817-6

**Published:** 2021-09-06

**Authors:** Valentina Gonzalez-Jaramillo, Jelena Guyer, Nora Luethi, Piotr Sobanski, Rut Zbinden, Eveline Rodriguez, Lukas Hunziker, Steffen Eychmüller, Maud Maessen

**Affiliations:** 1 Institute of Social and Preventive Medicine (ISPM), University of Bern, Bern, Switzerland; 2 Graduate School for Health Sciences, University of Bern, Bern, Switzerland; 3 Department of General Surgery, Spital Emmental, Burgdorf, Switzerland; 4 Faculty of Medicine, University of Bern, Bern, Switzerland; 5 University Center for Palliative Care, Inselspital, University Hospital Bern, University of Bern, Bern, Switzerland; 6 Department of Emergency Medicine, Inselspital, University Hospital Bern, University of Bern, Bern, Switzerland; 7 Palliative Care Unit and Competence Centre, Department of Internal Medicine, Spital Schwyz, Schwyz, Switzerland; 8 Stiftung Für Langzeitpflege, Tilia Köniz, Köniz, Switzerland; 9 Department of Cardiology, Inselspital University Hospital Bern, Bern, Switzerland

**Keywords:** Needs assessment, Heart failure, Palliative care, Patient-centered care, NAT: PD-HF

## Abstract

**Background:**

The Needs Assessment Tool: Progressive Disease-Heart Failure (NAT: PD-HF) is a tool created to assess the needs of people living with heart failure and their informal caregivers to assist delivering care in a more comprehensive way that addresses actual needs that are unmet, and to improve quality of life. In this study, we aimed to (1) Translate the tool into German and culturally adapt it. (2) Assess internal consistency, inter-rater reliability, and test–retest reliability of the German NAT: PD-HF. (3) Evaluate whether and how patients and health care personnel understand the tool and its utility. (4) Assess the tool’s face validity, applicability, relevance, and acceptability among health care personnel.

**Methods:**

Single-center validation study. The tool was translated from English into German using a forward–backward translation. To assess internal consistency, we used Cronbach´s alpha. To assess inter-rater reliability and test–retest reliability, we used Cohen´s kappa, and to assess validity we used face validity.

**Results:**

The translated tool showed good internal consistency. Raters were in substantial agreement on a majority of the questions, and agreement was almost perfect for all the questions in the test–retest analysis. Face validity was rated high by health care personnel.

**Conclusion:**

The German NAT: PD-HF is a reliable, valid, and internally consistent tool that is well accepted by both patients and health care personnel. However, it is important to keep in mind that effective use of the tool requires training of health care personnel.

**Supplementary Information:**

The online version contains supplementary material available at 10.1186/s12955-021-01817-6.

## Introduction

Heart failure (HF) is a global pandemic currently affecting at least 26 million people worldwide. Its prevalence is growing as the population ages and other risk factors increase [1]. Despite optimal recommended therapies, and apart from uncontrolled disease-related symptoms such as shortness of breath, pain, sleep disorders, and fatigue [2], people living with HF often suffer from other conditions such as depression and anxiety [3]. Moreover, taking care of or living with a person suffering from chronic HF can be stressful and burdensome [4]. Both patients with HF and their families are at increased risk of experiencing physical, emotional, and financial burdens that may impair their quality of life [5]. Failure to assess such burdens routinely and systematically in daily medical practice contributes to undertreatment and unnecessary suffering.

Needs assessment tools are clinical decision aids, facilitating the detection of patient needs and the assignment of actions to address them according to the available care options. These tools used as a support and a starting point for delivering patient-centered care [6]. Two recent systematic reviews found six needs measurement tools that can be used with patients with HF [6, 7]. Both systematic reviews found that the most comprehensive of these tools and the only one created specifically for patients with HF is the “Needs Assessment Tool: Progressive Disease-Heart Failure” (NAT: PD-HF) [8].

The NAT: PD-HF was developed in 2013 by Australian researchers to help health care personnel identify the needs of patients with HF and their informal caregivers, and match them with the most appropriate services regardless of whether they may be psychology, social work, cardiology, specialized palliative care, or general medicine [8]. The NAT: PD-HF, more than a questionnaire, is a direct, face-to-face interaction guide aimed to increase the attention dedicated to patients and their narratives in a highly efficient and effective way. Clinical environments that optimize this attention, will not only improve the quality of care and patient and staff satisfaction, but also reduce care costs [9, 10]. The implementation of the NAT: PD-HF in the clinical practice could be a fundamental basis for strategies focusing on optimizing attention.

To date the NAT: PD-HF has been available in only English [8] and Dutch [11]. We therefore (1) translated the tool into German and culturally adapted it, (2) assessed psychometric characteristics of the translated NAT: PD-HF that include internal consistency, inter-rater reliability, and test–retest reliability, (3) evaluated whether and how patients and health care personnel understood the tool and its utility, and (4) assessed the face validity, applicability, relevance, and acceptability of the German NAT: PD-HF among health care personnel.

## Methods

### Study design

We conducted this work at Inselspital, the University Hospital of Bern, Switzerland, a tertiary academic hospital with a dedicated HF clinic and a specialized palliative care service. Translation and validation of the original tool was performed in accordance with the guidelines of the European Organisation for Research and Treatment of Cancer [12]. We performed separate forward and backward translations (Additional file 1), and evaluated internal consistency, inter-rater reliability, and test–retest reliability.

We surveyed patients (Additional file 2) and health care personnel (Additional file 3) to gauge their level of understanding of the NAT: PD-HF and perception of its utility. Face validity, applicability, relevance, and acceptability of the tool by health care personnel were assessed with interviews of personnel specializing in cardiology and palliative care (PC) at our institution (Additional file 4). Sociodemographic and basic clinical information of the participants was extracted from electronic medical records.

### The original NAT: PD-HF tool

The NAT: PD-HF is a comprehensive tool designed for health care personnel to assess both a patient’s and a main informal caregiver’s physical, psychological, social, and spiritual needs [8]. It consists of three parts: the first one is a user guide explaining the purpose of the tool and how to complete it, the second is a questionnaire comprised of 20 items, and the third section has examples of what to address within each item of the questionnaire (Additional file 5). The questionnaire is subdivided into sections addressing four topics: (1) priority referral for further assessment, (2) patient well-being, (3) ability of the informal caregiver or family to care for the patient, and (4) caregiver well-being. For each question, health care personnel selects the level of concern together with the patient: (1) none, (2) some/potential, (3) significant. If there is some/potential or significant concern in one of the questions, the patient has unmet needs. The health care worker proceeds to choose an appropriate action: (1) directly manage it, (2) management by another care team member, or (3) refer. If the last option is chosen, the yellow box at the end of the questionnaire (under the heading: referral required for further assessment of care) should be filled in. In this section, there is the option to refer to other providers such as the patient’s general practitioner, social worker, psychologist, specialist PC service, cardiologist, or other.

### Translation and cultural adaptation

The NAT: PD-HF was translated from English to German using a forward–backward translation procedure and cultural adaptation by four translators, one of which was a professional medical text translator (Additional file 1). Cultural adaptation required minimal change to the third part of the NAT: PD-HF, which includes examples of what to address within each of the questionnaire items (Additional file 6). The second part of the NAT: PD-HF, the questionnaire itself, did not require any cultural adaptation. Therefore, we did not consider it necessary to validate the content of the culturally adapted version.

After pilot testing the translated tool with 19 patients, we made small adjustments to the translation, and in the recruitment process. The pilot test also revealed the need to train health care personnel who will use the tool. Because of the adjustments after the pilot test, we excluded pilot participants from the final analysis.

### Participants and recruitment

Eligible participants were adult patients (≥ 18 years of age) with an appointment at the Heart Failure Clinic at Inselspital Bern who had had at least one consultation in the clinic and who could fluently communicate in German. No specific stage or severity of HF was selected to ensure a representative, full spectrum of the disease.

Patients meeting the inclusion criteria received a study invitation package containing an invitation letter and study description, a participant information sheet and consent form, and a removable response card that patients could send back to the research team stating whether they were interested in taking part of the study or not. For those who were interested, we arranged an appointment with a study member immediately before or after their scheduled consultation at the HF clinic.

We evaluated patients' cognitive capacity by asking them three questions about the study after having explained its purpose and the content from the consent form. The questions were (1) what is the aim of the study? (2) In which patients will the study be performed? (3) As part of this study, will you have blood tests or ultrasounds? In case of satisfactory answers to the three questions, the interviewer proceeded with the signing of the consent form and the interview.

### Study procedure

#### Using the tool with HF patients

**Fig. 1:**
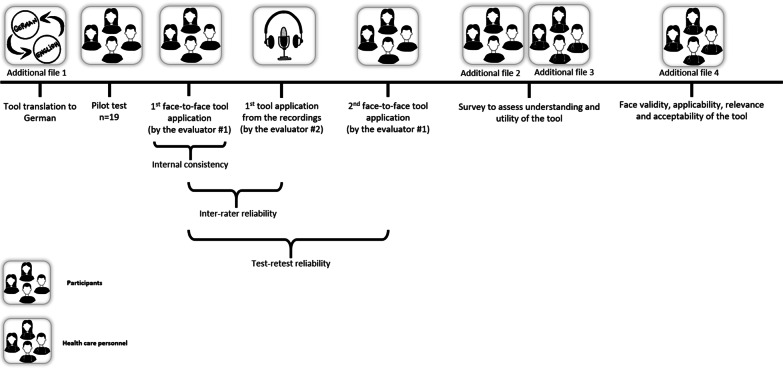
Study procedure. Adobe Illustrator Draw was used to create the artwork

Using the German version of the NAT: PD-HF a general practitioner trained in administering the tool directly queried the patient and, if present, an informal caregiver. From these data we estimated internal consistency (Fig. 1). The encounter was recorded to allow a second evaluator to provide data for gauging inter-rater reliability using the audio recording (Fig. 1). After 10 to 20 days, a second appointment was scheduled with the patient and the NAT: PD-HF was repeated face-to-face by the same evaluator who used the tool during the first assessment to provide data for gauging test–retest reliability (Fig. 1).

#### Assessing understanding of the German NAT: PD-HF and its utility

At the end of the study, we asked patients to tell us whether they thought the NAT: PD-HF questions were easy to understand and answer, and whether the questions might lead to better care. We obtained their answers via a five-point Likert scale ranging from ''strongly agree'' to ''strongly disagree'' (Additional file 2). We also asked about whether such questions are addressed in the course of their regular clinical consultations, and asked them what other questions they thought should be included in the tool.

We asked health care personnel representing all potential patient referral services to complete the same survey that patients completed (Additional file 3).

#### Face validity, applicability, relevance and acceptability

Interviews were conducted with the group of health care personnel to assess face validity, applicability, relevance, and acceptability of the German NAT: PD-HF (Additional file 4).

### Data analysis

#### Internal consistency and sample size

We used Cronbach’s alpha to assess internal consistency. Sample size calculations were based on Feldt´s formula [13]. To have 80% power at an alpha error of 5%, a lowest acceptable Cronbach´s alpha value of 0.75, and an expected value of 0.85, the sample size calculated was 66. Estimating a dropout rate of 10% we aimed to recruit 75 patients.

#### Inter-rater reliability and test–retest reliability

To assess the agreement between the results obtained from the physician using the NAT: PD-HF the first time and the results when the second evaluator filled out the tool listening the recordings, it is the inter-rater reliability, we used Cohen`s kappa. Since our data were ordinal, we used Cohen’s weighted kappa. According to Cohen, 1968, the investigators choose the weights based on their own judgment [14]. Weights should be defined so that a weight of 1 (a full weight) is assigned to diagonal agreements, whereas decreasing weights are assigned to partial agreements depending on the problem under investigation [15]. We defined weight in such a way that the difference between “No concern” and “Some/potential concern” is less than the difference between “No concern” and “Significant concern.” We similarly regarded the difference between “No concern” and “Some/potential concern” as greater than the difference between “Some/potential concern” and “Significant concern” since, in the second case, at least, the professionals agreed that some additional action should be taken. (The NAT: PD-HF instructions state that the professional using the tool should act on each identified need). We weighted the agreement of the two evaluators acknowledging these differences as presented in Additional file 7: Table 1.

The frequency of some/potential and significant concerns were low for some of the NAT: PD-HF items (Additional file 8: Table 2). We therefore decided to additionally report the results of the prevalence-adjusted and bias-adjusted kappa (PABAK) to avoid obtaining inflated agreements due to bias introduced by those low frequencies [16].

To assess the agreement between the results of the physician using the tool the first time and the results of the same physician using the tool the second time, it is the test–retest reliability, we estimated Cohen`s. For this calculation, we used the same weights presented in the Additional file 7: Table 1, as well as PABAK.

We interpreted inter-rater and test–retest reliabilities as near-perfect agreement if the kappa was greater than 0.81, as substantial if the kappa was between 0.61 and 0.80, moderate if it was between 0.41 and 0.60, and poor if it was less than 0.40 [17].

Unlike the first evaluator, the second evaluator was not always the same. Therefore, we did a sensitivity analysis to assess the inter-rater reliability for each one of the two second evaluators and define whether the data could be analyzed as a whole or if we needed to account for second evaluator differences.

#### Survey of patients and health care personnel

To summarize surveys results, “Strongly agree” and “Agree,” were pooled with each other and “Disagree” and “Strongly disagree” were pooled with each other. The original five-point Likert scale was thus converted into a three-point scale for the analysis. All analyses were performed using STATA release 15 (Stata Corp, College Station, Texas).

## Results

### Participants

**Fig. 2:**
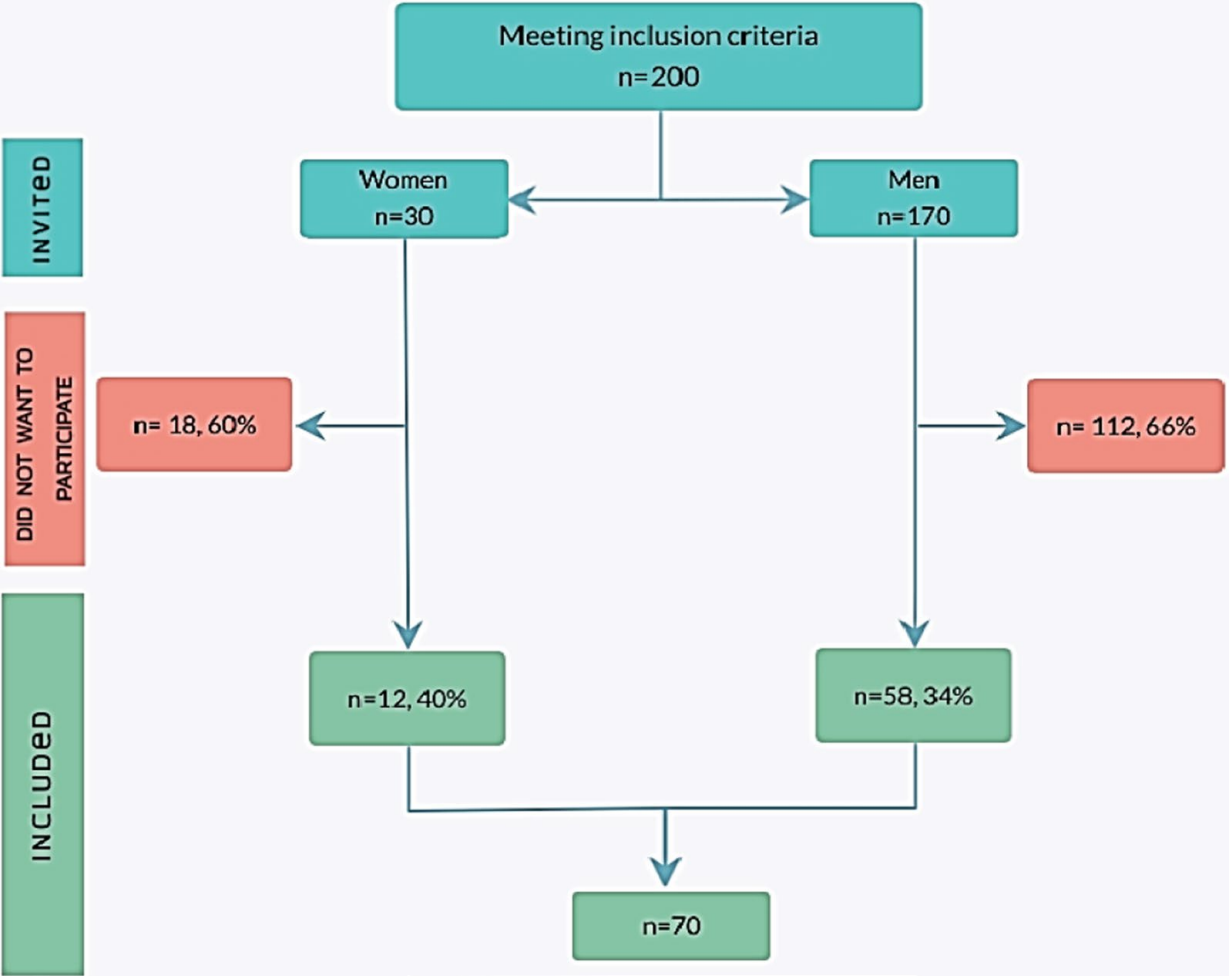
Flowchart of the participants included in the study

Between December 2019 and March 2020, 200 patients meeting the inclusion criteria were invited to participate. Among those invited, 70 patients consented to participate, giving a recruitment rate of 35% (Fig. 2). Though men predominated among the invitees, women (12/30, 40%) were slightly more willing to participate than men (58/170, 34%) (Fig. 2). There was no loss to follow-up.

A large majority of the patients included in the study were men (58/70, 83%); the mean age of all participants was 62.0 years (SD 13.7) (Table 1). The majority of the patients (45/70, 64%) had a reduced (≤ 40%) left ventricular ejection fraction (LVEF). Among the remaining 25 patients, three had a borderline (41–49%) LVEF, and the LVEF was preserved (≥ 50%) in 22. Half of the patients were classified as NYHA II, while none was classified as NYHA IV.

**Table 1:** General characteristics of the population included in the study

Sociodemographic characteristics	n (%) or median (IQR)
Women	12 (17%)
Age	62 (54–72)
*Marital status*	
Single	12 (17%)
Widowed	2 (3%)
Divorced	13 (19%)
Married/partnership	43 (61%)
*Religion*	
Protestant	37 (55%)
Roman catholic	19 (28%)
Non-denominational	11 (17%)
*Clinical characteristics*	
NYHA functional class	
I	22 (31%)
II	35 (50%)
III	13 (19%)
LVEF (%)	35 (20–50)
*Classification according to LVEF*	
HFrEF	45 (64%)
HFmrEF	3 (4%)
HFpEF	22 (32%)
ICD	38 (54%)
VAD	12 (17%)
Transplant list	9 (13%)
COPD	11 (16%)
CAD	30 (43%)
CKD	32 (46%)
*Palliative care-related characteristics*	
Presence of caregiver	67 (95%)
*Caregiver*	
Couple	49 (73%)
Son or daughter	6 (9%)
Sibling	4 (6%)
Parent	3 (4%)
Other	5 (8%)
Have requested referral to palliative care	0 (0.0%)
Have an advance directive	25 (36%)

*IQR *interquartile rate, *LVEF* left ventricular ejection fraction, *HFrEF* heart failure with reduced ejection fraction, *HFmrEF* heart failure with mildly reduced ejection fraction, *HFpEF* heart failure with preserved ejection fraction, *ICD* implantable cardioverter defibrillator, *VAD* ventricular assist device, *COPD* chronic obstructive pulmonary disease, *CAD* coronary artery disease, *CKD* chronic kidney disease

The interviews took an average of 24.1 min (SD 9.7) and the frequency of answers for each question is presented in Additional file 8: Table 2. Almost all the patients (67/70) reported the availability of an informal caregiver in case of need, the majority of whom are their partners (49/67, 73%). Patients were also asked about their need for more information about the course and prognosis of the disease and about treatment. Twenty-six reported a need for more information about one or more of seven aspects of living with HF; the largest need reported was that for information on legal and financial issues (Additional file 8: Table 2).

### Tool translation

The culturally adapted German translation of the NAT: PD-HF is presented in Additional file 1.

### Psychometric characteristics of the German NAT: PD-HF

#### Internal consistency

With a Cronbach’s alpha of 0.83, the internal consistency was high. Furthermore, when removing each question to evaluate how the alpha changed without each item Cronbach’s alpha ranged from 0.80 and 0.84. Details of the psychometric characteristics are presented in Table 2.

**Table 2:** Inter-rater and test–retest reliability

	Inter-rater reliability		Test–retest reliability	
	Cohen´s kappa	PABAK	Cohen´s kappa	PABAK
*Section 2: Patient wellbeing*				
1. Is the patient experiencing unresolved physical symptoms (including problems with breathlessness, pain, fatigue, nausea, edema, insomnia, or cough)?	0.43	0.49	0.94	0.94
2. Does the patient have problems with daily living activities?	0.59	0.79	0.99	0.98
3. Does the patient have psychological symptoms that are interfering with well-being or relationships?	0.69	0.76	0.94	0.94
4. Does the patient have concerns about how to manage his/her medication and treatment regimens?	0.48	0.94	1.00	1.00
5. Does the patient have concerns about spiritual or existential issues?	0.88	0.96	0.90	0.97
6. Does the patient have financial or legal concerns that are causing distress or require assistance?	0.85	0.89	0.93	0.94
7. From the health delivery point of view, are there health beliefs, cultural, or social factors involving the patient or family that are making care more complex?	0.17	0.80	0.90	0.97
*Section 3: Ability of caregiver or family to care for patient*				
1. Is the caregiver or family distressed about the patient’s physical symptoms?	0.83	0.98	0.83	0.98
2. Is the caregiver or family having difficulty providing physical care?	0.78	0.84	0.82	0.87
3. Is the caregiver or family having difficulty coping?	0.82	0.88	0.97	0.98
4. Is the caregiver having difficulty managing the patient’s medication and treatment regimens?	1.00	1.00	1.00	1.00
5. Does the caregiver or family have financial or legal concerns that are causing distress or require assistance?	0.66	0.87	1.00	1.00
6. Is the family currently experiencing problems that are interfering with their functioning or interpersonal relationships or is there a history of such problems?	0.57	0.79	0.96	0.97
*Section 4: Caregiver wellbeing*				
1. Is the caregiver or family experiencing physical, practical, spiritual, existential, or psychological problems that are interfering with their well-being or functioning?	0.73	0.89	0.85	0.93

*PABAK* prevalence-adjusted and bias-adjusted kappa

#### Inter-rater reliability

Overall, based on Cohen´s kappa 13 out of the 14 questions reached at least moderate agreement (defined as a kappa value ≥ 0.41). Three questions about patient´s wellbeing in section "2" had an almost perfect, three had substantial and only one had poor agreement. This last question, that is the last one of the section, aims to identify the health beliefs of patients and cultural or social factors that might be barriers to health delivery. Among the six questions assessing the ability of the caregiver to take care of the patient in section "3", three questions were in almost perfect agreement, two had substantial agreement, and the remaining one moderate agreement. The question from section "4" about the caregiver´s well-being had substantial agreement (Table 2).

We observed no differences in the performance of the different first and second evaluators (Additional file 9: Table 3). Therefore, there was no need to account for second evaluators’ differences in the analyses.

#### Test–retest reliability

The median time between the two appointments was 15 days (IQR 14–20) and none of the patients reported any significant change in his or her condition between the two appointments. Test–retest reliability of the German NAT: PD-HF was very high and all questions had almost perfect agreement, with Cohen`s kappa ranging from 0.82 to 1.00 (Table 2).

#### Survey of patients

All of the 70 participants agreed that the tool questions were easy to understand and answer. Sixty-one patients (87%) thought that discussing the issues raised in the questions may improve quality of care. Only three patients reported these questions being routinely asked or discussed during clinical consultations (Table 3).

**Table 3:** Results of the survey applied to patients and health care personnel

	Agree	Neither agree nor disagree	Disagree
*Survey to patients (n = 70)*			
1.The questions were, generally, easy to understand (n = 69)	69 (100%)		
2.The questions were, generally, easy to answer (n = 69)	69 (100%)		
3.If my doctor asks me these questions, it may help to improve the quality of my care (n = 68)	61 (90%)	6 (9%)	1 (1%)
4.The questions asked in the questionnaire are usually dealt with during the clinical consultation (n = 70)	3 (4%)	38 (54%)	29 (42%)
*Survey to health care personnel (n = 27)*			
1.In general, the questions were easy to understand for the patient (n = 27)	13 (48%)	14 (52%)	
2.In general, the questions were easy to answer for the patient (n = 27)	10 (37%)	16 (59%)	1 (4%)
3.The quality of the care is improved by applying this tool (n = 27)	23 (85%)	4 (15%)	
4.The questions asked in the questionnaire are usually dealt with during the clinical consultation (n = 25)	11 (44%)	14 (56%)	

Regarding other questions that should be included in the tool, the most frequently mentioned topics were lifestyle habits and sexuality. More specifically, patients suggested inquiry about exercise and nutrition habits, and whether there have been changes in a couple’s sex life and there are side effects of medication that interfere with it.

#### Survey of health care personnel

We interviewed 27 health care professionals. These included eight cardiologists, five cardiology nurses, four PC specialists, three PC nurses, three psychologists from the HF clinic, two general practitioners, and two social workers. Among the 27 interviewees, 21 were women, and 14 of the interviewees have worked in the profession for more than 10 years.

In general, the health care personnel rated the questions easy for patients to understand, but not to answer. Most (23 of the 27 professionals) considered the tool helpful for improving the quality of patient care. Eleven of the health care personnel reported that the questions in the tool are usually asked during a clinical consultation, while the remaining 16 responded in a neutral manner, neither agreeing nor disagreeing. The four PC specialists agreed that topics from the tool are discussed during a routine consultation with a PC specialist. In contrast to that, only two of the cardiologists said topics from the tool are discussed in consultations, while the remaining six said that most “are not addressed,” and that “psychosocial aspects are not discussed” (Table 3).

### Face validity, applicability, relevance and acceptability

#### Face validity

Face validity was rated high, with most of the health care personnel (23 of 27) agreeing that the tool appears to measure unmet needs of both patients with HF and their caregivers. The remaining four interviewees partially agreed, but thought that to properly assess caregiver needs the caregiver needs to be present in the interview (Table 4).

**Table 4:** Results of the interview to assess tool´s face validity, applicability, relevance and acceptability among health care personnel

	Agree	Neutral	Disagree	Comments to highlight
*Face validity (n = 27)*				
1) The tool measures unmet needs of patients with heart failure and their caregivers	23 (85%)	4 (15%)		“to properly assess caregiver needs, the caregiver should be present in the interview”, “the tool is more useful for younger patients as they may have a higher disease burden than older patients”
*Applicability (n = 27)*				
1) The tool is easy to use	17 (63%)	7 (26%)	3 (11%)	“very complex and detailed”, “difficult to differentiate between *Some/Potential* and *Significant* in the level of concern”
2) Different professional groups can fill out the tool	23 (85%)	4 (15%)		
2. a) Which professional group should fill out the tool?				Palliative care staff (nurses and physicians) replied: physicians and nurses; General practitioners and social workers replied: physicians, nurses, social worker; Psychologists replied: physicians and nurses Cardiology staff (nurses and physicians) replied: mainly nurses;
3) The tool instructions are was easy to understand	27 (100%)			
4) The tool instructions are helpful	25 (93%)	2 (7%)		"instructions are useful but too long"
5) A special training is necessary to fill out the tool	16 (59%)	1 (4%)	10 (37%)	"for certain items, explanations are required", "maybe not a training, but an introduction to explain the purpose of the tool to increase motivation", "online training is an option", "training is required for non-specialized nurses", "the first and the last page of the tool have a lot of information that can be explained during a training session"
6) There are some difficulties in using the tool	18 (67%)	4 (15%)	5 (18%)	"many questions involve several aspects", "regarding the field *action taken*, there are situations you cannot act", "patients’ needs sufficient linguistic and cognitive skills", "an entire clinic needs to be willing to use the tool", " time consuming, especially with patients who have a lot to say", "needs a lot of time and empathy", "if filled out by nurses, they are not allowed to refer patients"
*Relevance (n = 27)*				
2) Some questions are irrelevant and can be left out	1 (4%)	3 (11%)	23 (85%)	"3.6: it is not clear what the question intends to assess", "2.7: not very useful", "2.7: formulated very complicated and too long", “section 3" and "4" are repeated and should be merged”, “questions 2.5 and 2.3 have a similar content”, "2.5: not sure what it should capture"
*Acceptability (n = 27)*				
1) Filling out the tool does not take too much time and can be integrated into daily routine clinical practice	13 (48%)	5 (19%)	9 (33%)	"it is too long and takes too much time", "it requires training or a very complete introduction", "not possible in the cardiology consultation, maybe the palliative care has time"
a) When should the tool be applied?				"early, in the first or second consultation", "not too early, it needs a basis of trust", "not too early, when the patient has dealt with the disease", "if the patient becomes more symptomatic or consultations become more frequent"
b) How often should the tool be applied?				"when something has change", "at rehospitalisation", "regularly, every 6 months", "during annual controls", "in patients who are deteriorating faster", "after regular intervals of 6 -12 months ask those questions where there were needs", "ask in every consultation if something has changed"
2) I feel uncomfortable asking some of the questions	4 (15%)	9 (33%)	14 (52%)	"questions 2.3, 2.5, 2.6, 3.6 and 4.2 are about topics hard to discuss (emotional, spiritual concerns, family or financial issues)", "patient may not like to talk about financial issues (question 2.6)", "2.5 and 2.6 need to be asked carefully (spiritual concerns or financial issues)", "2.3 is very personal (psychological issues)"

#### Applicability

Among the 27 health care personnel, 17 (63%) consider the tool easy to use, and all but two consider the written instructions on use the tool helpful. However, some thought the tool and its instructions were too long and too detailed.

PC specialists, psychologists, and social workers think that doctors (general practitioners, cardiologists, PC specialists) as well as nurses can use the tool, while cardiology staff think that mainly nurses should use it (Table 4).

#### Relevance

Two PC specialists, one general practitioner and a psychologist, each questioned the relevance of some elements of the tool. One of the PC specialist was not sure what question 2.5 should cover. The other PC specialist said that question 2.7 is not very useful since it collapses different factors into a single question, and suggested that section three, which is about the ability of caregiver to care for the patient, and section four, about caregiver well-being, should be merged. The GP thought question 3.6 is not very clear and should be rephrased, and the psychologist thought that questions 2.3 and 2.5 have similar content (Table 4).

#### Acceptability

There was no consensus on either when or how often the NAT: PD-HF should be employed in clinical practice. Some believe that it should be used early, at the first or second consultation, while others believe that it is better not to use it early, but only after a basis of trust has been built with the interviewer and the patient has already dealt with the disease. Some respondents think it should be used at regular intervals such as every six months or at annual check-ups, while others think it should be used again after major changes in health status or after hospitalization (Table 4).

The main concerns about accepting the NAT: PD-HF are lack of time to use it, and that being able to use it requires some training or at least a very thorough introduction to using it and its value (Table 4).

Another reservation about using the NAT: PD-HF reported by cardiologists and cardiology nurses was that some staff members do not feel comfortable discussing emotional, spiritual, or financial issues with their patients (Table 4).

## Discussion

### Key results

We translated the NAT: PD-HF into German, culturally adapted, and validated it. The validation showed good internal consistency and substantial inter-rater agreement for the majority of the items. Additionally, we were able to assess the test–retest reliability and we found almost perfect agreement between the first and second assessments. Moreover, patients thought well of the tool, and they agreed that it could help to improve their quality of care and that it covered relevant topics that are not normally addressed in clinical consultations. Similarly, face validity and user-friendliness were rated highly by health care professionals. However, like the participating health care personnel we also believe training is necessary to ensure correct use of the tool.

### NAT: PD-HF strengths

Other such tools based on patient prognosis ignore the needs that patients with longer prognoses may have, and how difficult it is to predict disease trajectory in HF [18–20]. The NAT: PD-HF evaluates patient needs in a more integrated and profound way. It assesses not only patient well-being and information needs, but also the needs and well-being of family members and informal caregivers. Two recently published systematic reviews of tools to assess PC needs in patients with HF concluded that the NAT: PD-HF was the most appropriate tool to assess the needs of patients with HF [6, 7]. The English version of the tool had already demonstrated a good correlation between evaluators [8] and good acceptability by health professionals [8, 11]. Our study supports the previous findings and additionally shows that the NAT: PD-HF is stable over time and that patients regard it as useful.

### NAT: PD-HF limitations

We observed no consensus across medical staff on when to use the tool for the first time, nor on how often to use it. However, a recently published position statement from the European Association of Palliative Care proposed an algorithm for when to employ the NAT: PD-HF. The association also recommended assessing PC needs in patients in less advanced stages of HF at annual reviews or after any significant health-related event for those in more advanced stages [21].

Although the tool was designed to be used without training, experience with the staff in this study showed that prior training is necessary. Training was mainly on how to discuss the needs of the patients and the informal caregiver in an empathic, respectful and efficient way by applying situation-specific adaptations of the example sentences of the instruction manual and on how to decide on cut-off points of the scoring system. In a similar study assessing the NAT: PD-HF in the Netherlands, nurses trained in using the tool still requested they referred that more training to assess PC needs [11]. A qualitative study that evaluated barriers in the implementation of the Needs Assessment Tool: Progressive Disease-Interstitial Lung Disease (NAT: PD-ILD), also found the need for training for the correct application of that tool [22].

The majority of the questions had a substantial inter-rater agreement. In section "2", question number seven had a Cohen´s kappa of 0.17, which is a poor correlation. This finding is consistent with the validation of the English version of the NAT: PD-HF and the NAT: PD-Cancer [8, 23]. The poor correlation might be the result of aiming to assess multiple factors in one question. The question, aiming to identify barriers for the health care delivery, tries to assess social factors, health beliefs, and cultural beliefs from both the patient and the family. One rater may focus more on one factor than on others, leading to obtaining different answers from patients. In section "3", question number six had the lowest kappa of the section. The difficulties discussing delicate topics such as interpersonal problems in the family might explain the low kappa. Additionally, this question also aims to assess multiple issues within the same question.

### Implications for future research

Its high face validity suggests that the translated tool appears to measure unmet needs of patients with HF and their informal caregivers, and both medical staff and patients think the tool could help to improve quality of care. However, its effectiveness in reducing unmet needs and consequently even improving quality of life has not yet been studied. It would be interesting to prospectively study the effectiveness of this tool in reducing unmet needs and increasing quality of life in clinical practice.

Additionally, this study aimed to assess the psychometric characteristics of the German NAT: PD-HF in a representative full-spectrum of HF patients. However, the psychometric profile of the tool might differ according to characteristics such as NYHA functional class, patient gender, or the availability of a caregiver. A study assessing these differences should take into account the sample size needed to make such subgroup analyses.

### Implications for clinical practice

Although we tried to include patients from across the disease spectrum, most patients were male and had reduced ejection fraction. Patients with preserved ejection fraction are more often women, are older, and have more comorbidities (and therefore more symptoms) such as diabetes, hypertension, and renal dysfunction [24, 25]. In addition, drugs that have shown to improve symptoms in patients with reduced ejection fraction have shown little or no effectiveness in patients with preserved ejection fraction [26]. Therefore, the latter patients might have different types of needs than patients with reduced ejection fraction. However, this study did not seek to assess the needs in patients with HF but rather to evaluate the psychometric characteristics of the tool. Hence, we believe that the low proportion of patients with preserved ejection fraction and women does not limit the generalizability of our results to clinical practice. However, we acknowledge that, due to the low proportion of women in our study (17%), we do not know the acceptability and women´s opinion of the German NAT: PD-HF. Similarly, due to the lack of participation of patients at NYHA IV, we do not know their acceptance of the tool, which could be low due to the cognitive challenge that answering this questionnaire could pose for these patients.

More than one-third of the patients cited a need for further information. During interviews some staff also mentioned that they did not feel comfortable discussing certain topics. These reports from patients and staff together suggest that improving doctor-patient communication may assist using the NAT: PD-HF more effectively in clinical practice to recognize unmet needs and improve daily life for people living with HF. As attention is linked to higher-quality communication, one way to improve communication would be to adjust the conditions of clinical practice so that patients and their narratives receive sufficient attention for the physician to fully understand their situation or need [9].

## Conclusion

The German NAT: PD-HF is a reliable, valid, and internally consistent tool that is well accepted by both patients and health care personnel. However, it is important to keep in mind that effective use of the tool requires training of health care personnel.

## Supplementary Information

**Additional file 1**. German version of the “Needs Assessment Tool: Progressive Disease-Heart Failure (NAT: PD-HF)”. Instrument zur Erfassung der Bedürfnisse: progressive Erkrankung - Herzinsuffizienz (IEB: PE-HI).

**Additional file 2**. Template of the survey to patients.

**Additional file 3**. Template of the survey to health care personnel.

**Additional file 4**. Template of the interview to assess face validity, applicability, relevance and acceptability of the tool among health care personnel.

**Additional file 5**. Original version (English) of the “Needs Assessment Tool: Progressive Disease-Heart Failure (NAT: PD-HF)”.

**Additional file 6**. Changes made for the cultural adaptation.

**Additional file 7**. **Table 1.** Matrix of the weights used to assess inter-rater reliability and test-retest reliability.

**Additional file 8**. **Table 2.** Frequency of answers from the first application of the tool.

**Additional file 9**. **Table 3.** Sensitivity analysis to assess the inter-rater reliability for each one of second evaluators.

## Data Availability

The datasets used and/or analysed during the current study are available from the corresponding author on reasonable request.

## Abbreviations

HF: Heart failure
NAT: PD-HF: Needs assessment tool: progressive disease-heart failure
PC: Palliative care

## Code availability

Do file with coding for statistical analyses performed in STATA release 15 (Stata Corp, College Station, Texas), will be available upon request to the corresponding author.
