# Supplementary material for: Validation of the German version of the needs assessment tool: progressive disease-heart failure
Source: Health Qual Life Outcomes. 2021 Sep 6;19:214. doi: 10.1186/s12955-021-01817-6 (PMC8419951; doi:10.1186/s12955-021-01817-6)
Supplement: Supplementary file 1 — Additional file 1. German version of the “Needs Assessment Tool: Progressive Disease-Heart Failure (NAT: PD-HF)”. Instrument zur Erfassung der Bedürfnisse: progressive Erkrankung - Herzinsuffizienz (IEB: PE-HI). [file 12955_2021_1817_MOESM1_ESM.docx]

## **Additional file 1.** German version of the “Needs Assessment Tool: Progressive Disease-Heart Failure (NAT: PD-HF)”. Instrument zur Erfassung der Bedürfnisse: progressive Erkrankung - Herzinsuffizienz (IEB: PE-HI)

**Instrument zur Erfassung der Bedürfnisse: progressive Erkrankung - Herzinsuffizienz (IEB:PE-HI)**Benutzerleitfaden

| **Zweck des IEB:PE-HI** |
| --- |
| - Das Instrument zur Erfassung der Bedürfnisse: Progressive Erkrankung - Herzinsuffizienz (IEB:PE-HI) wird sowohl im allgemeinmedizinischen als auch im spezialisierten Bereich verwendet. Es kann dazu beitragen, die Art und den Umfang der Bedürfnisse von Menschen mit Herzinsuffizienz und ihren Betreuungspersonen mit den geeigneten Personen oder Diensten abzustimmen, um auf diese Bedürfnisse einzugehen. - Im allgemeinmedizinischen Bereich (z. B. Hausarztpraxis und Kardiologie) kann das IEB:PE-HI verwendet werden, um zu eruieren, welcher Bedarf in diesem Umfeld gedeckt werden kann und welche Bedürfnisse komplexer sind und eher in die Hände anderer Fachpersonen gehören. - Im spezialisierten Bereich (z. B. spezialisierte Palliativdienste) kann das IEB:PE-HI bei der Feststellung komplexer Bedürfnisse helfen und als Instrument zur Koordination der Betreuung nach einer Hospitalisierung oder zur Ermittlung des Bedarfs nach weiterer Unterstützung dienen. - Das IEB:PE-HI ist ein wichtiges Instrument zur Erleichterung der Kommunikation zwischen Leistungserbringern der Grundversorgung und der spezialisierten Versorgung über Patientenbedürfnisse und zu deren Deckung ergriffene Massnahmen. |

| **Ausfüllen des IEB:PE-HI** |
| --- |
| Das IEB:PE-HI ist ein Instrument zur Erfassung der Bedürfnisse, das von Gesundheitsfachpersonen auf zahlreichen Fachgebieten verwendet werden kann. Beim Ausfüllen des IEB:PE-HI sollte wie folgt vorgegangen werden:   1. BEWERTEN Sie FÜR JEDEN PUNKT die Besorgnis des Patienten bzw. der Betreuungsperson, und zwar mithilfe der Optionen: «Keine», «Mittel/potenziell» und «Erheblich». 2. BEDENKEN Sie bei jedem Thema die diversen Fragen, die für die Person in der aktuellen Krankheitsphase relevant sind. Als Grundlage können Sie die separate Anleitung verwenden. 3. HANDELN Sie bei jedem Bedürfnis, das besorgniserregend ist («Mittel/potenziell» und «Erheblich»). Ihre Massnahmen können umfassen: direkt von Ihnen zu erledigen, von einem anderen Mitglied des professionellen Behandlungsteams zu erledigen oder Überweisung an eine Person ausserhalb des Behandlungsteams. Halten Sie Ihre Massnahmen auf dem IEB:PE-HI fest. 4. ÜBERWEISEN Sie die Person im Bedarfsfall, indem Sie den diesbezüglichen Abschnitt am Ende des Fragebogens ausfüllen. Stellen Sie dabei sicher, dass die Angaben über die Stelle, an die überwiesen wird, die Priorität der Überweisung und den Kenntnisstand des Betroffenen über die Überweisung vollständig sind. 5. INFORMIEREN Sie die anderen Mitglieder des Behandlungsteams über die Ergebnisse der Bedürfniserfassung durch:    1. Einfügen einer Kopie des IEB:PE-HI in die Patientenakte.    2. Übermitteln einer Kopie an den Hausarzt, Kardiologen bzw. einen anderen Facharzt, der die betroffene Person betreut.    3. Weiterleiten einer Kopie an die Überweisungsstelle (falls Überweisung erforderlich). 6. BEWERTEN Sie die Bedürfnisse ERNEUT, indem Sie das IEB:PE-HI ungefähr im Monatsabstand oder bei einer Änderung des funktionellen Status des Patienten bzw. der Betreuungsperson ausfüllen.   Anmerkung: Zur besseren Lesbarkeit wird jeweils nur die männliche Form verwendet; gemeint sind jedoch immer beide Geschlechter. |

| Instrument zur Erfassung der Bedürfnisse: progressive Erkrankung-herzinsuffizienz (IEB:PE-HI)  Bitte alle abschnitte ausfüllen  Name des Patienten/der Patientin:_______________________________________ Datum:____________ | PATIENTEN-/ADRESSEN- ETTIKETTE | | |
| --- | --- | --- | --- |
| **Abschnitt 1. P**rioritäre Überweisung zur weiteren Abklärung | | | |
|  | Ja | Nein | Falls Felder mit Punkt angekreuzt wurden: Ziehen Sie eine Abklärung durch einen spezialisierten Palliativdienst in Betracht. |
| 1. Steht dem Patienten im Bedarfsfall eine Betreuungsperson zur Verfügung? |  | * |  |
| 2. Hat der Patient oder die Betreuungsperson um die Überweisung an einen spezialisierten Palliativdienst gebeten? | * |  |  |
| 3. Benötigen Sie Unterstützung bei der Organisation der Betreuung des Patienten und/oder seiner Betreuungsperson? | * |  |  |

| **Abschnitt 2.** Wohlbefinden des Patienten (weitere Hinweise: siehe Anleitung auf der nächsten Seite) | | | | | | | | | |
| --- | --- | --- | --- | --- | --- | --- | --- | --- | --- |
|  | | | Grad der Besorgnis | | | Zu ergreifende Massnahmen | | | |
|  |  |  | Keine | Potenziell / Mittel | Erheblich | Direkt zu  erledigen | | Von anderem Mitglied des Behandlungsteams zu erledigen | Überweisung erforderlich |
| 1. Hat der Patient unkontrollierten körperliche Symptome (z.B. Atemnot, Schmerzen, Müdigkeit, Übelkeit, Ödeme, Schlaflosigkeit, oder Husten)? | | |  |  |  |  | |  |  |
| 2. Hat der Patient Probleme bei alltäglichen Tätigkeiten? | | |  |  |  |  | |  |  |
| 3. Hat der Patient psychologische Symptome, die das Wohlbefinden oder die Beziehungen beeinträchtigen? | | |  |  |  |  | |  |  |
| 4. Hat der Patient Schwierigkeiten beim Umgang mit seinen Medikamenten und dem Behandlungsplan? | | |  |  |  |  | |  |  |
| 5. Ist der Patient über spirituelle oder existenzielle Fragen besorgt? | | |  |  |  |  | |  |  |
| 6. Beschäftigen den Patienten finanzielle oder juristische Fragen, die ihn belasten oder bei denen er Unterstützung benötigt? | | |  |  |  |  | |  |  |
| 7. Gibt es vom Standpunkt der Gesundheitsversorgung gesehen vonseiten des Patienten und/oder der Angehörigen Vorstellungen über Gesundheit oder kulturelle oder soziale Faktoren, durch die die Betreuung komplexer wird? | | |  |  |  |  | |  |  |
| 8. Benötigt der Patient Informationen über:  (Zutreffendes ankreuzen) | Die Prognose | Behandlungsmöglichkeiten | Patientenverfügung / lebensverlängernde Massnahmen | | | | | Finanzielle / juristische Fragen | |
|  |  | Herzkrankheit | Medizin- / Gesundheits- /  Unterstützungsdienste | | | | Beziehungsfragen/ psychologische Fragen | | |

ANMERKUNGEN:__________________________________________________________________________

| **Abschnitt 3.** Fähigkeit der Betreuungsperson, für den Patienten zu sorgen (weitere Hinweise: siehe Anleitung auf der nächsten Seite) | | | | | | | | | |
| --- | --- | --- | --- | --- | --- | --- | --- | --- | --- |
| Von wem stammen diese Angaben? (Zutreffendes ankreuzen)  Patient  Betreuungsperson  Beide | | | Grad der Besorgnis | | | | Zu ergreifende Massnahmen | | |
|  |  |  | Keine | | Potenziell / Mittel | Erheblich | Direkt zu  erledigen | Von anderem Mitglied des Behandlungsteams zu erledigen | Überweisung erforderlich |
| 1. Wird die Betreuungsperson durch die körperlichen Symptome des Patienten belastet?? | | |  | |  |  |  |  |  |
| 2. Hat die Betreuungsperson Schwierigkeiten bei der alltäglichen körperlichen Versorgung des Patienten? | | |  | |  |  |  |  |  |
| 3. Wird die Betreuungsperson durch die psychologischen Symptome des Patienten belastet? | | |  | |  |  |  |  |  |
| 4. Hat die Betreuungsperson Schwierigkeiten beim Umgang mit den verordneten Medikamenten und dem Behandlungsplan des Patienten? | | |  | |  |  |  |  |  |
| 5. Beschäftigen die Betreuungsperson finanzielle oder juristische Fragen, die sie belasten oder bei denen sie Unterstützung benötigt? | | |  | |  |  |  |  |  |
| 6. Haben die Angehörigen derzeit Probleme, die Konflikte verursachen oder die zwischenmenschlichen Beziehungen beeinträchtigen, oder bestanden solche Probleme in der Vergangenheit? | | |  | |  |  |  |  |  |
| 7. Benötigt die Betreuungsperson Informationen über:  (Zutreffendes ankreuzen) | Die Prognose | Behandlungsmöglichkeiten | | Patientenverfügung / lebensverlängernde Massnahmen | | | | Finanzielle / juristische Fragen | |
|  | Verhalten im Falle des Ablebens des Patienten | Herzkrankheit | | Medizin- / Gesundheits- /  Unterstützungsdienste | | | | Beziehungsfragen/ psychologische Fragen | |

ANMERKUNGEN: _________________________________________________________________________

| **Abschnitt 4.** Wohlbefinden der Betreuungsperson (weitere Hinweise: siehe Anleitung auf der nächsten Seite) | | | | | | |
| --- | --- | --- | --- | --- | --- | --- |
| Von wem stammen diese Angaben? (Zutreffendes ankreuzen)  Patient  Betreuungsperson  Beide | Grad der Besorgnis | | | Zu ergreifende Massnahmen | | |
|  | Keine | Potenziell / Mittel | Erheblich | Direkt zu erledigen | Von anderem Mitglied des Behandlungsteams zu erledigen | Überweisung erforderlich |
| 1. Hat die Betreuungsperson körperliche, praktische, spirituelle, existenzielle oder psychologische Probleme, die ihr Wohlbefinden beeinträchtigen oder die Alltagsbewältigung erschweren? |  |  |  |  |  |  |
| 2. Empfindet die Betreuungsperson Trauer aufgrund des bevorstehenden oder kürzlich geschehenen Ablebens des Patienten, sodass ihr Wohlbefinden oder ihre Alltagsbewältigung darunter leidet? |  |  |  |  |  |  |

ANMERKUNGEN: ________________________________________________________________________

| **Falls zur weiteren Abklärung oder Behandlung Überweisung nötig: bitte diesen Abschnitt ausfüllen** |
| --- |
| 1. Überweisung an: (Name)  2. Überweisung an: (Fachgebiet)  Allgemeinmedizin    Sozialarbeit   Psychologie   Kardiologie    Spezialisierten Palliativdienst  Andere: _______________________________________  3. Priorität der Abklärung:  Dringend (innert 24 h)  Halb dringlich (2–7 Tage)  Nicht dringend (sobald möglich)  4. Überweisung mit dem/der Betroffenen besprochen:  Ja  Nein  5. Betroffene(r) stimmt Überweisung zu:  Ja  Nein  6. Überweisung von: Name: ___________________________________________  Funktion:____________________________________ Unterschrift: _________________________________________ |

**Anleitung: wichtige Fragen bei der Bewertung des Besorgnis Grads**

| **Abschnitt 2.** Wohlbefinden des Patienten |
| --- |
| 1. Körperliche Symptome   - Präsentiert sich der Patient mit unkontrollierten körperlichen Symptome, etwa Schläfrigkeit, Müdigkeit, Atembeschwerden, Übelkeit, Erbrechen, andauernder Husten, Schmerzen, Ödeme, Verstopfung, Schlafstörungen, Appetitverlust oder reduzierter Leistungsfähigkeit?   2. Alltagstätigkeiten   - Hat der Patient Schwierigkeiten beim Toilettengang, Duschen, Baden oder der Essenszubereitung? - Gibt es eine Betreuungsperson, die ihm dabei hilft?   3. Psychologische Aspekte   - Leidet der Patient an anhaltenden Stimmungstiefs, Traurigkeit, Schuldgefühlen, Reizbarkeit, Verlust der Freude oder des Interesses an bisher üblichen Tätigkeiten? - Empfindet der Patient Besorgnis, Anspannung, Wut, Angst, Nervosität, Hoffnungslosigkeit oder Isolation? - Äussert der Patient den Wunsch nach einem raschen Tod?   4. Medikamente und Behandlung   - Ist der Patient in der Lage, mit komplexen Medikationen und Behandlungen umzugehen?   5. Spirituelles und Existentielles   - Fühlt sich der Patient isoliert oder hoffnungslos? - Empfindet der Patient, dass das Leben sinnlos ist oder dass er das Leben vergeudet hat? - Benötigt der Patient Unterstützung, um geeignete spirituelle Ressourcen oder Dienste zu finden?   6. Finanzielles und Juristisches   - Gibt es finanzielle Fragen im Zusammenhang mit Einkommensverlust, Behandlungskosten, Reiseausgaben oder Materialbedarf? - Ist die Familie sozioökonomisch benachteiligt? - Gibt es Meinungsverschiedenheiten zwischen dem Patienten und der Betreuungsperson über juristische Fragen wie Behandlungsmöglichkeiten am Lebensende (end-of-life decisions) und vorausschauende Behandlungsplanung (advance care planning)? - Kennt der Patient die verfügbaren Finanzierungsmöglichkeiten und benötigt er Unterstützung, diese in Anspruch zu nehmen?   7. Überzeugungen über Gesundheit, kulturelle oder soziale Faktoren   - Haben der Patient bzw. die Betreuungsperson Überzeugungen oder Haltungen, die die Gesundheitsversorgung erschweren (z.B. ein Verbot für bestimmte Behandlungen)? - Gibt es Sprachschwierigkeiten? Ist ein Dolmetscher nötig? - Fühlt sich der Patient bzw. die Betreuungsperson gesellschaftlich isoliert? - Hat der Patient Schwierigkeiten, die medizinische Grundversorgung zu erreichen und in Anspruch zu nehmen? (z.B. Organisation des Transports, kein Hausarzt, kein Vertrauen zum Hausarzt) - Hat der Patient Migrationshintergrund und fühlt sich schlecht integriert? - Ist der Patient über 75 Jahre alt? (Anmerkung: Ältere Patienten sind bei spezialisierten Palliativdiensten unterrepräsentiert.)   8. Information   - Möchte der Patient mehr Informationen über den Verlauf und die Prognose der Krankheit und die Behandlungsmöglichkeiten?   Kennt der Patient die verschiedenen (Pflege-)Dienste, die ihm als Unterstützung zur Verfügung stehen, oder benötigt er Hilfe, um sie in Anspruch zu nehmen (z. B. finanzielle und juristische Unterstützung, psychologische Dienste, Selbsthilfegruppen, Seelsorge)? |
| **Abschnitt 3.** Fähigkeit der Betreuungsperson, für den Patienten zu sorgen |
| Mit Betreuungsperson ist diejenige Person gemeint, die den Patienten im Alltag am meisten unterstützt. (z.B. Partner/in, Angehörige, Nachbar/in, Pflegeperson, Haushälter/in). Bei einigen Fragen können damit auch mehrere Personen gemeint sein (z.B. alle nahen Angehörigen).  1. Körperliche Betreuung   - Hat die Betreuungsperson Schwierigkeiten mit alltäglichen Tätigkeiten oder praktischen Fragen wie medizinische Ausrüstung, Mobilität und Transport? |
| 2. Körperliche Symptome   - Belasten die körperlichen Symptome des Patienten die Betreuungsperson?   3. Psychologische Aspekte   - Hat die Betreuungsperson Schwierigkeiten, mit den psychologischen Symptomen des Patienten umzugehen? - Äussert die Betreuungsperson den Wunsch nach einem raschen Tod des Patienten?   4. Medikamente und Behandlung   - Hat die Betreuungsperson Schwierigkeiten beim Umgang mit komplexen Medikationen und dem Behandlungsplan?   5. Finanzielles und Juristisches   - Gibt es finanzielle Fragen im Zusammenhang mit Einkommensverlust, Behandlungskosten, Reiseausgaben oder Materialbedarf? - Ist die Familie sozioökonomisch benachteiligt? - Gibt es Meinungsverschiedenheiten zwischen dem Patienten und der Betreuungsperson über juristische Fragen wie die Verweigerung lebensverlängernder Massnahmen und die Patientenverfügung? - Kennt die Betreuungsperson die verfügbaren Finanzierungsmöglichkeiten und benötigt Sie Unterstützung, diese in Anspruch zu nehmen?   6. Angehörige und Beziehungen   - Gibt es zwischen dem Patienten und den Angehörigen Kommunikationsprobleme oder Konflikte wegen der Prognose, der Behandlungsmöglichkeiten oder der Verteilung der Rollen bei der Pflege? - Ist der Patient insbesondere über die Folgen der Krankheit für die Betreuungsperson bzw. die Angehörigen besorgt?   7. Information   - Möchte die Betreuungsperson mehr Informationen, etwa über den Verlauf und die Prognose der Krankheit und die Behandlungsmöglichkeiten? - Kennt die Betreuungsperson die verschiedenen (Pflege-)Dienste, die ihr als Unterstützung zur Verfügung stehen, oder benötigt sie Hilfe, um sie in Anspruch zu nehmen (z. B. Kurzzeitpflege, finanzielle und juristische Unterstützung, psychologische Dienste, Selbsthilfegruppen, Seelsorge)? |
| **Abschnitt 4.** Wohlbefinden der Betreuungsperson |
| 1. Körperliches und Psychosoziales   - Hat die Betreuungsperson körperliche Symptome, etwa Müdigkeit, körperliche Belastung, Blutdruck- und Herz-Kreislauf-Störungen, eine stressbedingte Krankheit oder Schlafstörungen? - Fühlt sich die Betreuungsperson deprimiert, verzweifelt, verängstigt, nervös, angespannt, wütend, reizbar oder verärgert gegenüber Dritten oder von der Situation überfordert? - Hat die Betreuungsperson relevante spirituelle oder existenzielle Probleme?   2. Verlust und Trauer (vor und nach Todesfall)   - Erlebt die Betreuungsperson intrusive Bilder, plötzliche Emotionen? Bestreiten sie, dass sich der Verlust auf sie auswirkt, und vernachlässigen sie nötige Anpassungen zu Hause oder im Beruf? |

## **Additional file 2.** Template of the survey to patients

|  | Strongly agree | Agree | Neither agree nor disagree | Disagree | Strongly disagree |
| --- | --- | --- | --- | --- | --- |
| The questions were, generally, easy to understand. |  |  |  |  |  |
| The questions were, generally, easy to answer. |  |  |  |  |  |
| If my doctor asks me these questions, it may help to improve the quality of my care. |  |  |  |  |  |
| The questions asked in the questionnaire are usually dealt with during the clinical consultation. |  |  |  |  |  |
| Bearing in mind that the purpose of the questionnaire is to identify unmet needs:  Do you think are there other questions we should include in the questionnaire? | | | | | |

## **Additional file 3.** Template of the survey to health care personnel

|  | Strongly agree | Agree | Neither agree nor disagree | Disagree | Strongly disagree |
| --- | --- | --- | --- | --- | --- |
| In general, the questions were easy to understand for the patient. |  |  |  |  |  |
| In general, the questions were easy to answer for the patient. |  |  |  |  |  |
| The quality of the care is improved by applying this tool. |  |  |  |  |  |
| The questions asked in the questionnaire are usually dealt with during the clinical consultation. |  |  |  |  |  |

## **Additional file 4.** Template of the interview to assess face validity, applicability, relevance and acceptability of the tool among health care personnel

Face validity: Needs Assessment Tool: Progressive disease – Heart failure (NAT: PD-HF)

Interview code­­­­___________

Date____________ Time___________

| General characteristics of the staff | |  |
| --- | --- | --- |
| Professional category  [physician](https://dict.leo.org/englisch-deutsch/physician) cardiology  physician palliative care  nurse cardiology  nurse palliative care  psychologist  social worker  general practitioner  others (e.g. medical student) | sex  female  male  professional experience  < 5  5-10  > 10 | |

| Interview | | | |
| --- | --- | --- | --- |
| **Face validity** | Agree | Neutral | Disagree |
| 1) The tool measures unmet needs of patients with heart failure and their caregivers   - comment: |  |  |  |
| **Applicability** | Agree | Neutral | Disagree |
| 1) The tool is easy to use   - comment: |  |  |  |
| 2) Different professional groups can fill out the tool   - comment:   2.a Which professional group should fill out the tool? |  |  |  |
| 3) The tool instructions are easy to understand |  |  |  |
| 4) The tool instructions are helpful |  |  |  |
| 5) A special training is necessary to fill out the tool |  |  |  |
| 6) There are some difficulties in using the tool   - If yes, which: |  |  |  |
| **Relevance** | Agree | Neutral | Disagree |
| 1) Some questions are irrelevant and can be left out   - If yes, which ones |  |  |  |
| **Acceptability** | Agree | Neutral | Disagree |
| 1) Filling out the tool does not take too much time and can be integrated into daily routine clinical practice  1.a) When should the tool be applied?  1.b) How often should the tool be applied? |  |  |  |
| 2) I feel uncomfortable asking some of the questions   - If yes, which and why: |  |  |  |

## **Additional file 5.** Original version (English) of the “Needs Assessment Tool: Progressive Disease-Heart Failure (NAT: PD-HF)”.


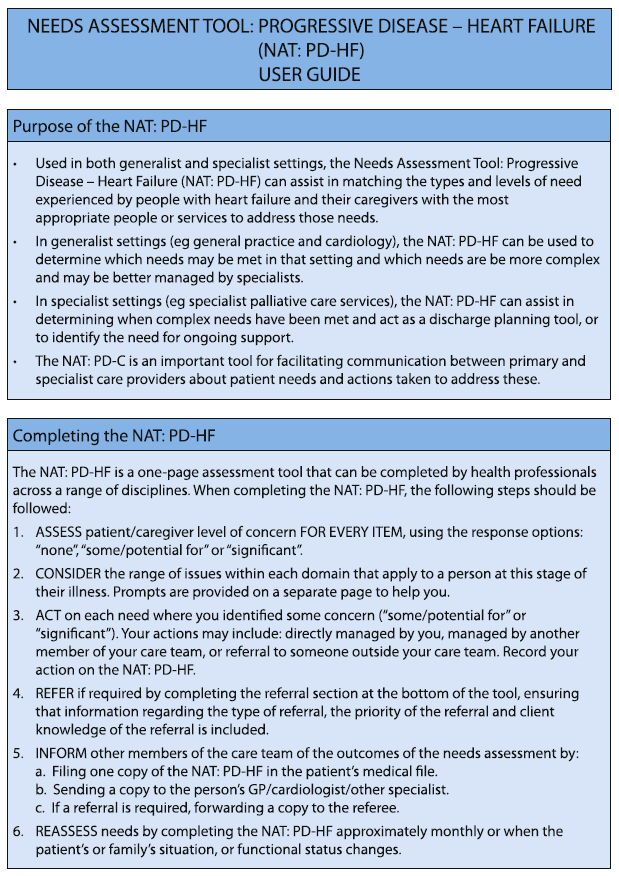


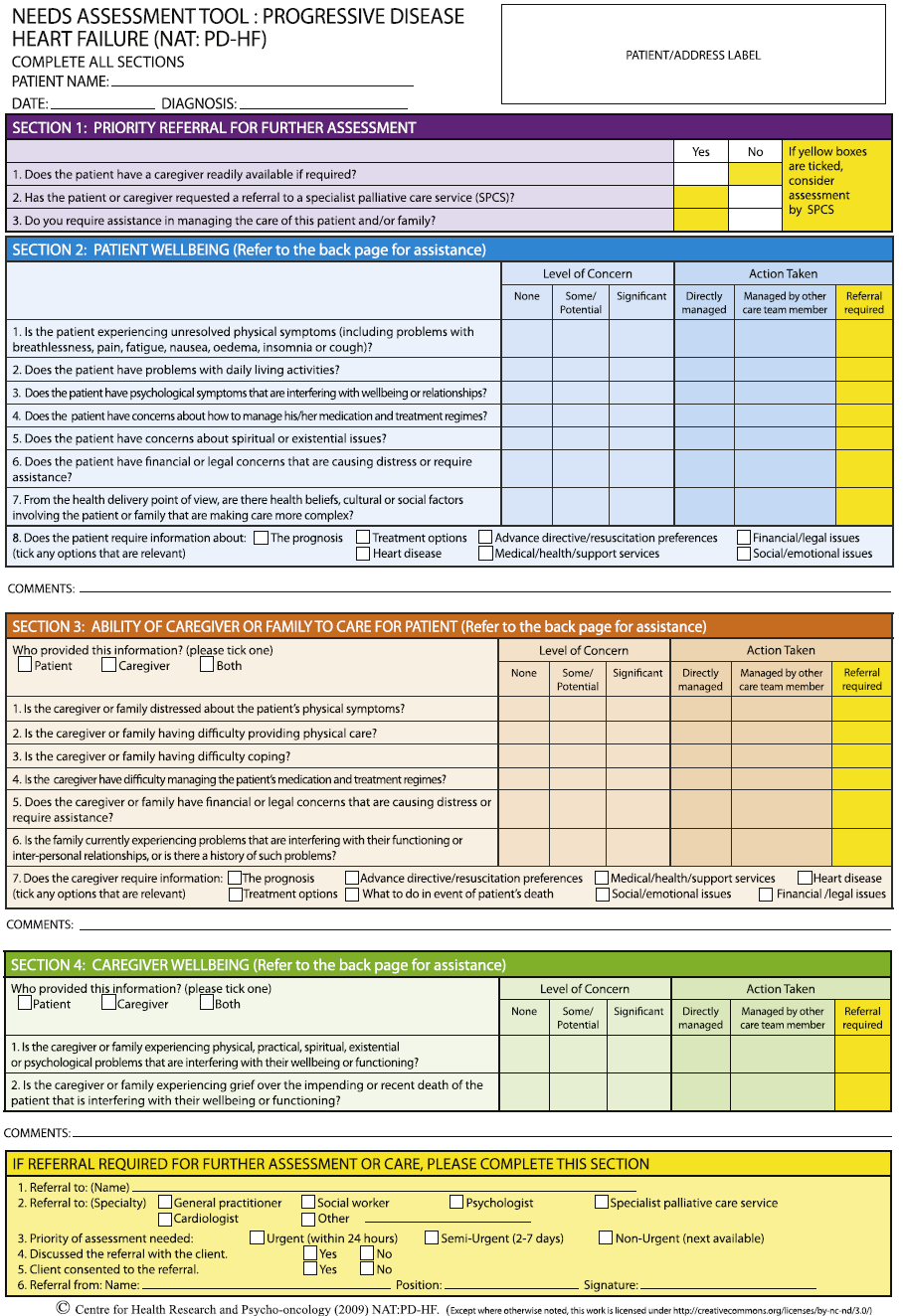


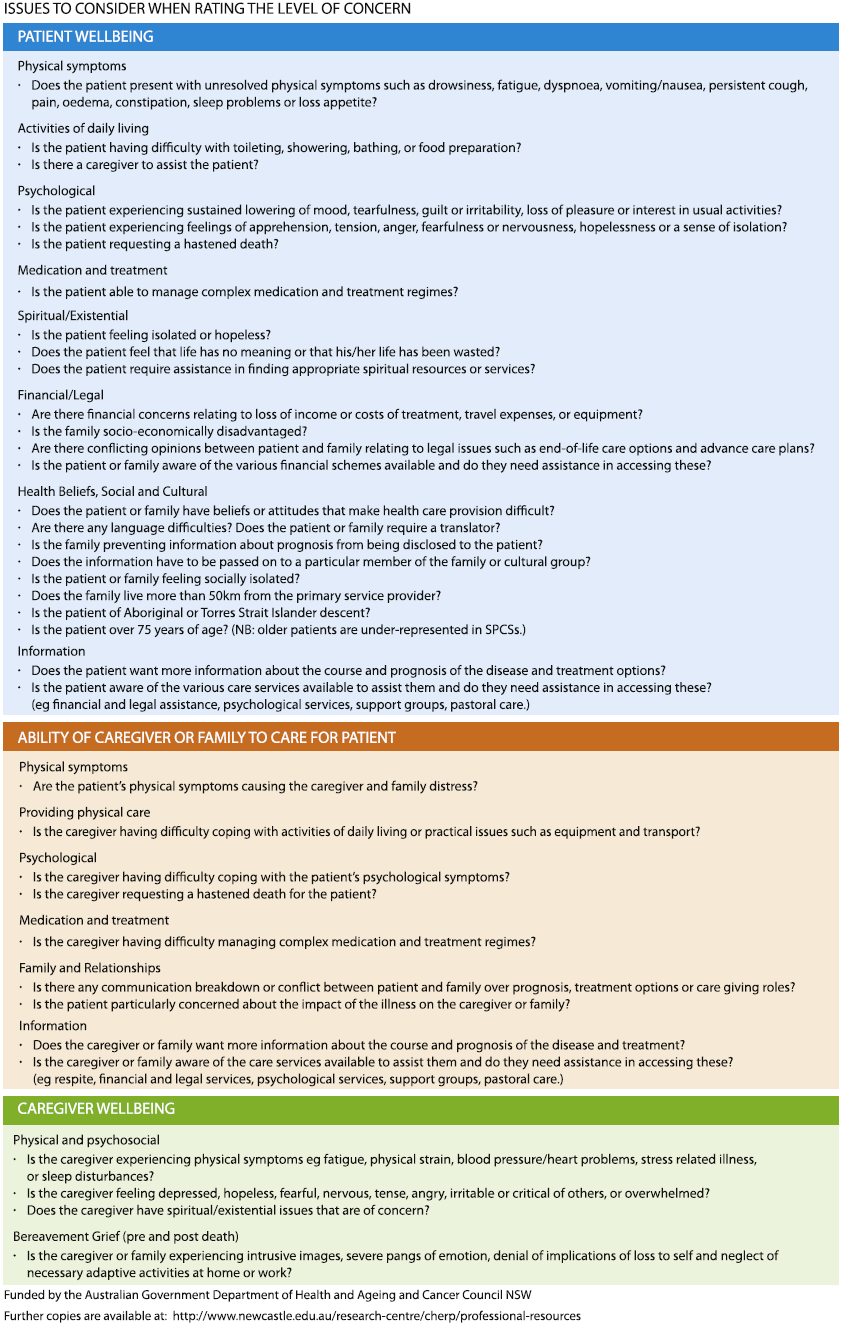


## **Additional file 6.** Changes made for the cultural adaptation


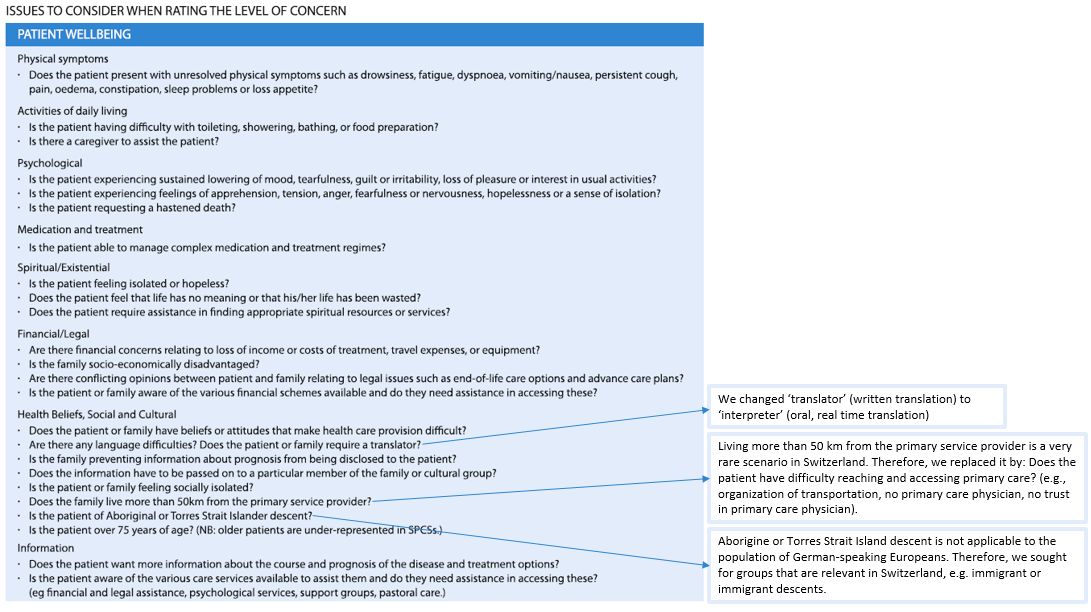


## **Additional file 7. Table 1.** Matrix of the weights used to assess inter-rater reliability and test-retest reliability

|  | No concern | Some/potential concern | Significant concern |
| --- | --- | --- | --- |
| No concern | 1 | 0.2 | 0 |
| Some/potential concern | 0.2 | 1 | 0.8 |
| Significant concern | 0 | 0.8 | 1 |

## **Additional file 8. Table 2.** Frequency of answers from the first application of the tool

|  | None | Some/potential | Significant |
| --- | --- | --- | --- |
| **Section 2. Patient wellbeing (n=70)** | | | |
| 1. Is the patient experiencing unresolved physical symptoms (including problems with breathlessness, pain, fatigue, nausea, edema, insomnia, or cough)? | 28 (40%) | 38 (54%) | 4 (6%) |
| 2. Does the patient have problems with daily living activities? | 60 (86%) | 9 (13%) | 1 (1%) |
| 3. Does the patient have psychological symptoms that are interfering with well-being or relationships? | 40 (57%) | 30 (43%) | 0 (0%) |
| 4. Does the patient have concerns about how to manage his/her medication and treatment regimens? | 67 (96%) | 3 (4%) | 0 (0%) |
| 5. Does the patient have concerns about spiritual or existential issues? | 64 (91%) | 6 (9%) | 0 (0%) |
| 6. Does the patient have financial or legal concerns that are causing distress or require assistance? | 51 (73%) | 19 (27%) | 0 (0%) |
| 7. From the health delivery point of view, are there health beliefs, cultural, or social factors involving the patient or family that are making care more complex? | 64 (91%) | 6 (9%) | 0 (0%) |
| **Section 3. Ability of caregiver or family to care for patient (n=67)** | | | |
| 1. Is the caregiver or family distressed about the patient’s physical symptoms? | 66 (99%) | 1 (1%) | 0 (0%) |
| 2. Is the caregiver or family having difficulty providing physical care? | 43 (64%) | 24 (36%) | 0 (0%) |
| 3. Is the caregiver or family having difficulty coping? | 49 (73%) | 18 (27%) | 0 (0%) |
| 4. Is the caregiver having difficulty managing the patient’s medication and treatment regimens? | 67 (100%) | 0 (0%) | 0 (0%) |
| 5. Does the caregiver or family have financial or legal concerns that are causing distress or require assistance? | 58 (87%) | 9 (13%) | 0 (0%) |
| 6. Is the family currently experiencing problems that are interfering with their functioning or interpersonal relationships or is there a history of such problems? (n=70) | 55 (79%) | 15 (21%) | 0 (0%) |
| **Section 4. Caregiver wellbeing (n=67)** | | | |
| 1. Is the caregiver or family experiencing physical, practical, spiritual, existential, or psychological problems that are interfering with their well-being or functioning? | 59 (88%) | 8 (12%) | 0 (0%) |
|  |  |  |  |
| **Yes** | **No** |  |  |
| **Does the patient require information about: (n=70)** |  |  |  |
| Heart disease | 3 (4%) | 67 (96%) |  |
| Treatment options | 4 (6%) | 66 (94%) |  |
| Financial/legal issues | 16 (23%) | 54 (77%) |  |
| Living will, life-extending measures | 6 (9%) | 64 (91%) |  |
| Prognosis | 3 (4%) | 67 (96%) |  |
| Medical/health/support services | 5 (7%) | 65 (93%) |  |
| Social/emotional issues | 2 (3%) | 68 (97%) |  |

## **Additional file 9. Table 3.** Sensitivity analysis to assess the inter-rater reliability for each one of second evaluators.

|  | Cohen´s kappa for second evaluator #1 | Cohen´s kappa for second evaluator #2 | higher kappa obtained by: |
| --- | --- | --- | --- |
| **Section 2. Patient wellbeing** | | | |
| 1. Is the patient experiencing unresolved physical symptoms  (including problems with breathlessness, pain, fatigue, nausea,  edema, insomnia, or cough)? | 0.38 | 0.46 | evaluator #2 |
| 2. Does the patient have problems with daily living activities? | 0.58 | 0.58 | equal |
| 3. Does the patient have psychological symptoms that are interfering with well-being or relationships? | 0.66 | 0.68 |  |
| 4. Does the patient have concerns about how to manage his/her medication and treatment regimens? | 1.00 | 0.48 | evaluator #1 |
| 5. Does the patient have concerns about spiritual or existential issues? | 0.88 | 0.88 | equal |
| 6. Does the patient have financial or legal concerns that are causing distress or require assistance? | 0.83 | 0.85 | evaluator #2 |
| 7. From the health delivery point of view, are there health beliefs, cultural, or social factors involving the patient or family that are making care more complex? | 0.00 | 0.20 | evaluator #2 |
| **Section 3. Ability of caregiver or family to care for patient** | | | |
| 1. Is the caregiver or family distressed about the patient’s physical symptoms? | 1.00 | 0.70 | evaluator #1 |
| 2. Is the caregiver or family having difficulty providing physical care? | 0.77 | 0.77 | equal |
| 3. Is the caregiver or family having difficulty coping? | 1.00 | 0.72 | evaluator #1 |
| 4. Is the caregiver having difficulty managing the patient’s medication and treatment regimens? | 1.00 | 1.00 | equal |
| 5. Does the caregiver or family have financial or legal concerns that are causing distress or require assistance? | 1.00 | 0.51 | evaluator #1 |
| 6. Is the family currently experiencing problems that are interfering with their functioning or interpersonal relationships or is there a history of such problems? | 0.86 | 0.44 | evaluator #1 |
| **Section 4. Caregiver wellbeing** | | | |
| 1. Is the caregiver or family experiencing physical, practical, spiritual, existential, or psychological problems that are interfering with their well-being or functioning? | 0.71 | 0.80 | evaluator #2 |
