# Supplementary material for: Validation of the German version of the needs assessment tool: progressive disease-heart failure
Source: Health Qual Life Outcomes. 2021 Sep 6;19:214. doi: 10.1186/s12955-021-01817-6 (PMC8419951; doi:10.1186/s12955-021-01817-6)
Supplement: Supplementary file 2 — Additional file 2. Template of the survey to patients. [file 12955_2021_1817_MOESM2_ESM.docx]

## **Additional file 2.** Template of the survey to patients

|  | Strongly agree | Agree | Neither agree nor disagree | Disagree | Strongly disagree |
| --- | --- | --- | --- | --- | --- |
| The questions were, generally, easy to understand. |  |  |  |  |  |
| The questions were, generally, easy to answer. |  |  |  |  |  |
| If my doctor asks me these questions, it may help to improve the quality of my care. |  |  |  |  |  |
| The questions asked in the questionnaire are usually dealt with during the clinical consultation. |  |  |  |  |  |
| Bearing in mind that the purpose of the questionnaire is to identify unmet needs:  Do you think are there other questions we should include in the questionnaire? | | | | | |
