# Supplementary material for: Validation of the German version of the needs assessment tool: progressive disease-heart failure
Source: Health Qual Life Outcomes. 2021 Sep 6;19:214. doi: 10.1186/s12955-021-01817-6 (PMC8419951; doi:10.1186/s12955-021-01817-6)
Supplement: Supplementary file 3 — Additional file 3. Template of the survey to health care personnel. [file 12955_2021_1817_MOESM3_ESM.docx]

## **Additional file 3.** Template of the survey to health care personnel

|  | Strongly agree | Agree | Neither agree nor disagree | Disagree | Strongly disagree |
| --- | --- | --- | --- | --- | --- |
| In general, the questions were easy to understand for the patient. |  |  |  |  |  |
|  |  |  |  |  |  |
|  |  |  |  |  |  |
| The questions asked in the questionnaire are usually dealt with during the clinical consultation. |  |  |  |  |  |
