# Supplementary material for: Validation of the German version of the needs assessment tool: progressive disease-heart failure
Source: Health Qual Life Outcomes. 2021 Sep 6;19:214. doi: 10.1186/s12955-021-01817-6 (PMC8419951; doi:10.1186/s12955-021-01817-6)
Supplement: Supplementary file 4 — Additional file 4. Template of the interview to assess face validity, applicability, relevance and acceptability of the tool among health care personnel. [file 12955_2021_1817_MOESM4_ESM.docx]

**Additional file 4.** Template of the interview to assess face validity, applicability, relevance and acceptability of the tool among health care personnel

Face validity: Needs Assessment Tool: Progressive disease – Heart failure (NAT: PD-HF)

Interview code­­­­___________

Date____________ Time___________

| General characteristics of the staff | |  |
| --- | --- | --- |
| Professional category  [physician](https://dict.leo.org/englisch-deutsch/physician) cardiology  physician palliative care  nurse cardiology  nurse palliative care  psychologist  social worker  general practitioner  others (e.g. medical student) | sex  female  male  professional experience  < 5  5-10  > 10 | |

| Interview | | | |
| --- | --- | --- | --- |
| **Face validity** | Agree | Neutral | Disagree |
| 1) The tool measures unmet needs of patients with heart failure and their caregivers   - comment: |  |  |  |
| **Applicability** | Agree | Neutral | Disagree |
| 1) The tool is easy to use   - comment: |  |  |  |
| 2) Different professional groups can fill out the tool   - comment:   2.a Which professional group should fill out the tool? |  |  |  |
| 3) The tool instructions are easy to understand |  |  |  |
| 4) The tool instructions are helpful |  |  |  |
| 5) A special training is necessary to fill out the tool |  |  |  |
| 6) There are some difficulties in using the tool   - If yes, which: |  |  |  |
| **Relevance** | Agree | Neutral | Disagree |
| 1) Some questions are irrelevant and can be left out   - If yes, which ones |  |  |  |
| **Acceptability** | Agree | Neutral | Disagree |
| 1) Filling out the tool does not take too much time and can be integrated into daily routine clinical practice  1.a) When should the tool be applied?  1.b) How often should the tool be applied? |  |  |  |
| 2) I feel uncomfortable asking some of the questions   - If yes, which and why: |  |  |  |
