# Supplementary material for: Validation of the German version of the needs assessment tool: progressive disease-heart failure
Source: Health Qual Life Outcomes. 2021 Sep 6;19:214. doi: 10.1186/s12955-021-01817-6 (PMC8419951; doi:10.1186/s12955-021-01817-6)

## **Additional file 5.** Original version (English) of the “Needs Assessment Tool: Progressive Disease-Heart Failure (NAT: PD-HF)”.


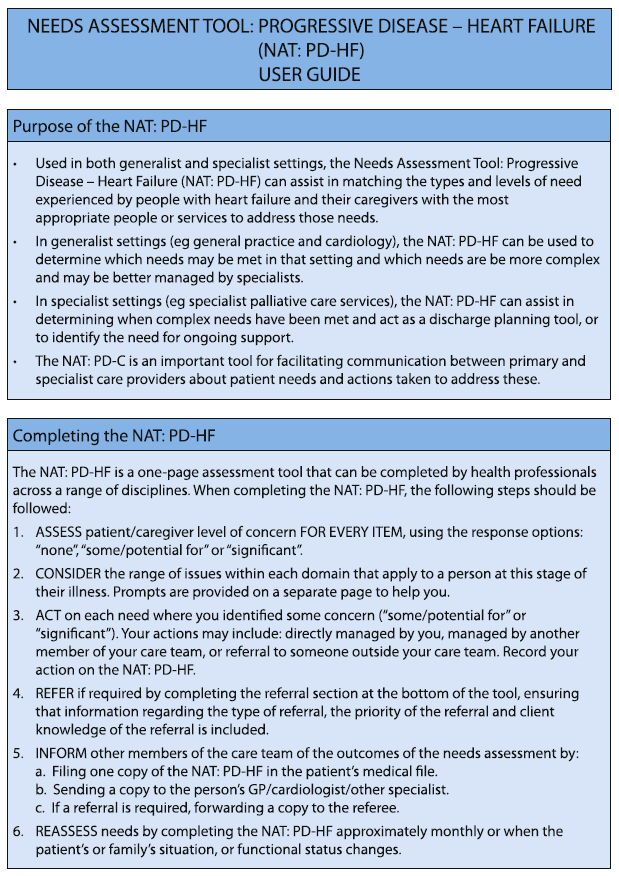


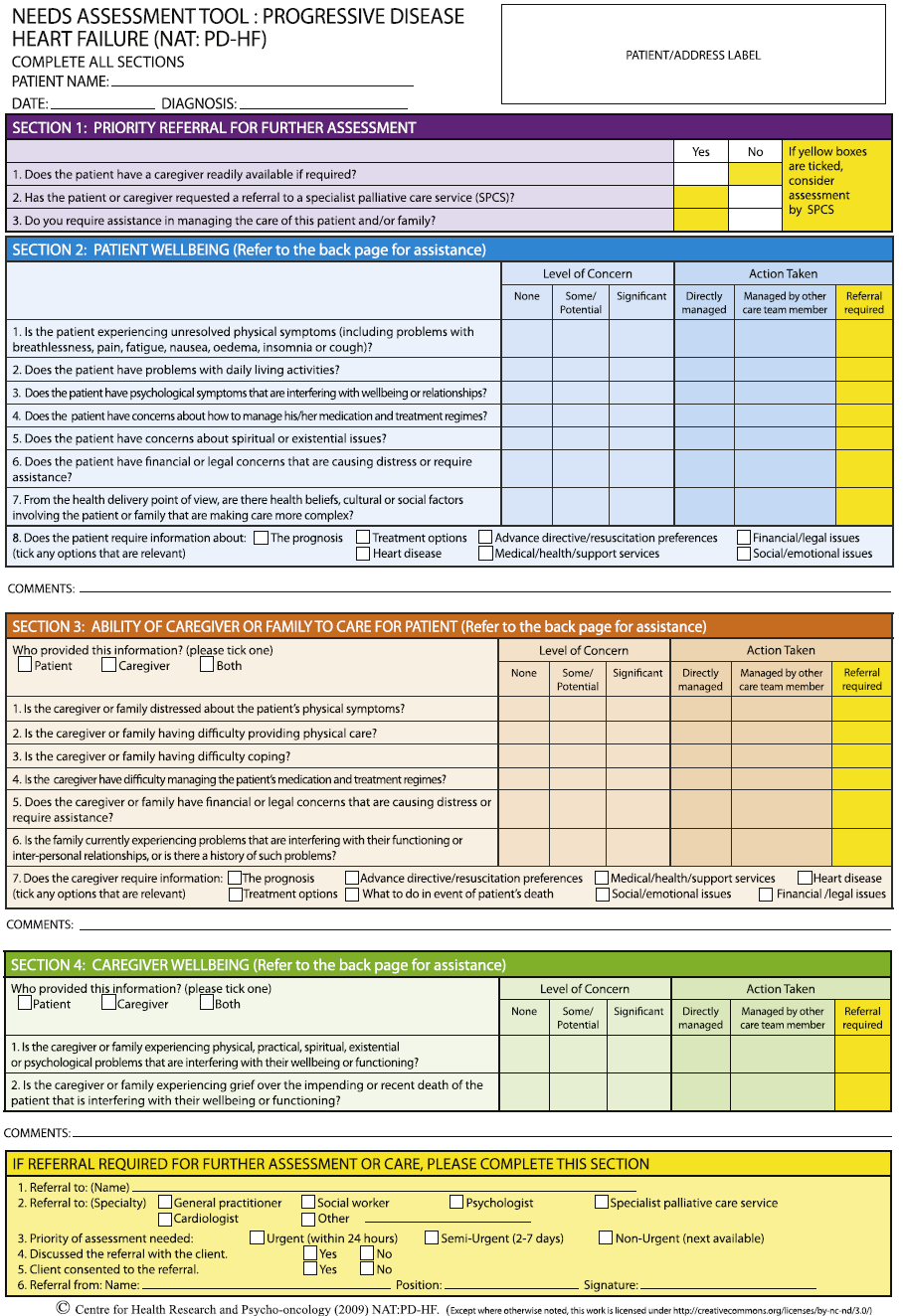


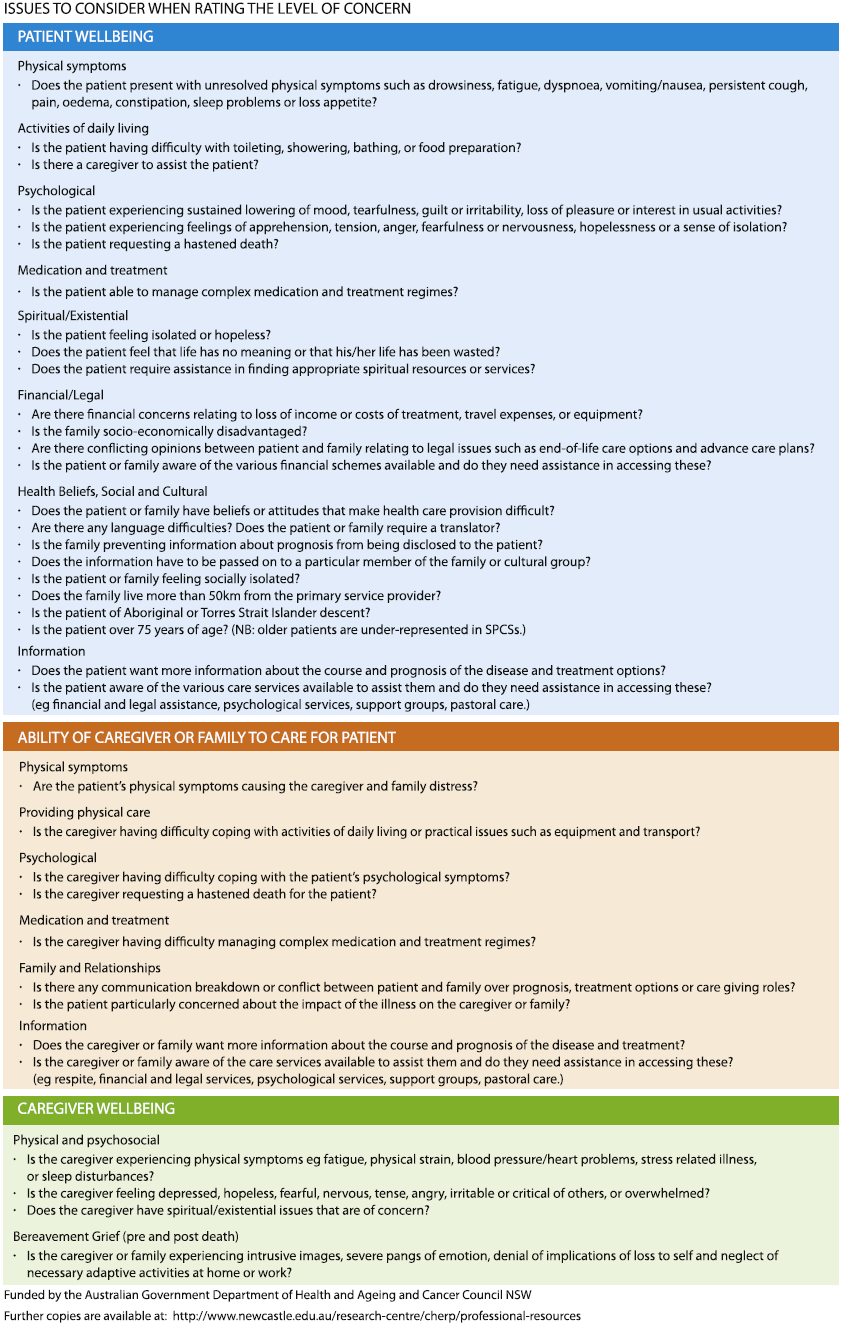

Supplement: Supplementary file 5 — Additional file 5. Original version (English) of the “Needs Assessment Tool: Progressive Disease-Heart Failure (NAT: PD-HF)”. [file 12955_2021_1817_MOESM5_ESM.docx]
