# Supplementary figures and images for: Validation of the German version of the needs assessment tool: progressive disease-heart failure
Source: Health Qual Life Outcomes. 2021 Sep 6;19:214. doi: 10.1186/s12955-021-01817-6 (PMC8419951; doi:10.1186/s12955-021-01817-6)

## **Additional file 6.** Changes made for the cultural adaptation


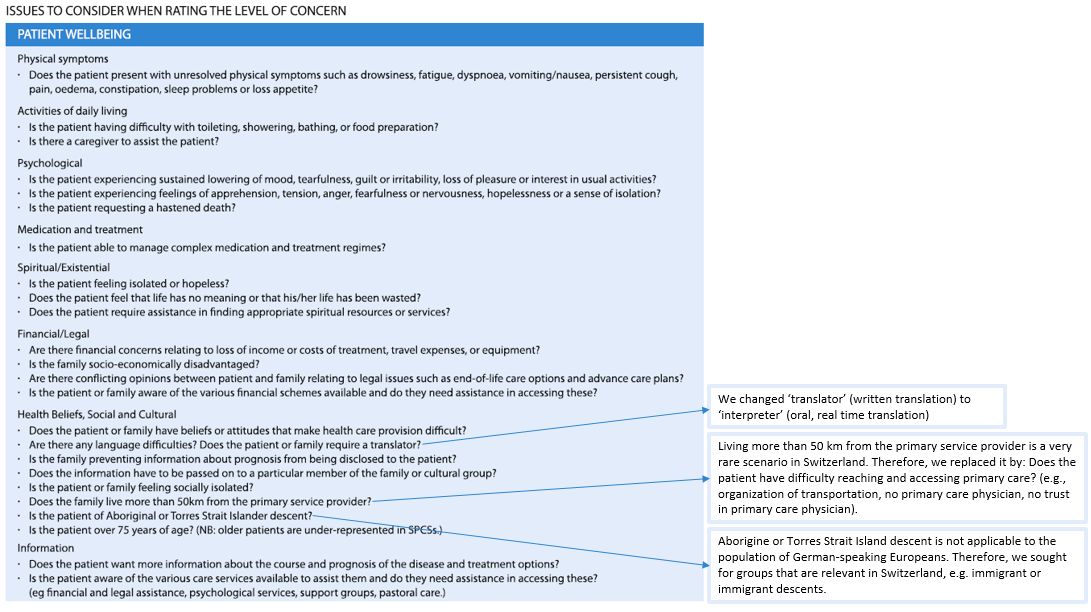

Supplement: Supplementary file 6 — Additional file 6. Changes made for the cultural adaptation. [file 12955_2021_1817_MOESM6_ESM.docx]
