# Supplementary material for: Validation of the German version of the needs assessment tool: progressive disease-heart failure
Source: Health Qual Life Outcomes. 2021 Sep 6;19:214. doi: 10.1186/s12955-021-01817-6 (PMC8419951; doi:10.1186/s12955-021-01817-6)
Supplement: Supplementary file 7 — Additional file 7. Table 1. Matrix of the weights used to assess inter-rater reliability and test-retest reliability. [file 12955_2021_1817_MOESM7_ESM.docx]

## **Additional file 7. Table 1.** Matrix of the weights used to assess inter-rater reliability and test-retest reliability

|  | No concern | Some/potential concern | Significant concern |
| --- | --- | --- | --- |
| No concern | 1 | 0.2 | 0 |
| Some/potential concern | 0.2 | 1 | 0.8 |
| Significant concern | 0 | 0.8 | 1 |
