# Supplementary material for: Validation of the German version of the needs assessment tool: progressive disease-heart failure
Source: Health Qual Life Outcomes. 2021 Sep 6;19:214. doi: 10.1186/s12955-021-01817-6 (PMC8419951; doi:10.1186/s12955-021-01817-6)
Supplement: Supplementary file 8 — Additional file 8. Table 2. Frequency of answers from the first application of the tool. [file 12955_2021_1817_MOESM8_ESM.docx]

## **Additional file 8. Table 2.** Frequency of answers from the first application of the tool

|  | None | Some/potential | Significant |
| --- | --- | --- | --- |
| **Section 2. Patient wellbeing (n=70)** | | | |
| 1. Is the patient experiencing unresolved physical symptoms (including problems with breathlessness, pain, fatigue, nausea, edema, insomnia, or cough)? | 28 (40%) | 38 (54%) | 4 (6%) |
| 2. Does the patient have problems with daily living activities? | 60 (86%) | 9 (13%) | 1 (1%) |
| 3. Does the patient have psychological symptoms that are interfering with well-being or relationships? | 40 (57%) | 30 (43%) | 0 (0%) |
| 4. Does the patient have concerns about how to manage his/her medication and treatment regimens? | 67 (96%) | 3 (4%) | 0 (0%) |
| 5. Does the patient have concerns about spiritual or existential issues? | 64 (91%) | 6 (9%) | 0 (0%) |
| 6. Does the patient have financial or legal concerns that are causing distress or require assistance? | 51 (73%) | 19 (27%) | 0 (0%) |
| 7. From the health delivery point of view, are there health beliefs, cultural, or social factors involving the patient or family that are making care more complex? | 64 (91%) | 6 (9%) | 0 (0%) |
| **Section 3. Ability of caregiver or family to care for patient (n=67)** | | | |
| 1. Is the caregiver or family distressed about the patient’s physical symptoms? | 66 (99%) | 1 (1%) | 0 (0%) |
| 2. Is the caregiver or family having difficulty providing physical care? | 43 (64%) | 24 (36%) | 0 (0%) |
| 3. Is the caregiver or family having difficulty coping? | 49 (73%) | 18 (27%) | 0 (0%) |
| 4. Is the caregiver having difficulty managing the patient’s medication and treatment regimens? | 67 (100%) | 0 (0%) | 0 (0%) |
| 5. Does the caregiver or family have financial or legal concerns that are causing distress or require assistance? | 58 (87%) | 9 (13%) | 0 (0%) |
| 6. Is the family currently experiencing problems that are interfering with their functioning or interpersonal relationships or is there a history of such problems? (n=70) | 55 (79%) | 15 (21%) | 0 (0%) |
| **Section 4. Caregiver wellbeing (n=67)** | | | |
| 1. Is the caregiver or family experiencing physical, practical, spiritual, existential, or psychological problems that are interfering with their well-being or functioning? | 59 (88%) | 8 (12%) | 0 (0%) |
|  |  |  |  |
| **Yes** | **No** |  |  |
| **Does the patient require information about: (n=70)** |  |  |  |
| Heart disease | 3 (4%) | 67 (96%) |  |
| Treatment options | 4 (6%) | 66 (94%) |  |
| Financial/legal issues | 16 (23%) | 54 (77%) |  |
| Living will, life-extending measures | 6 (9%) | 64 (91%) |  |
| Prognosis | 3 (4%) | 67 (96%) |  |
| Medical/health/support services | 5 (7%) | 65 (93%) |  |
| Social/emotional issues | 2 (3%) | 68 (97%) |  |
