# Supplementary material for: Validation of the German version of the needs assessment tool: progressive disease-heart failure
Source: Health Qual Life Outcomes. 2021 Sep 6;19:214. doi: 10.1186/s12955-021-01817-6 (PMC8419951; doi:10.1186/s12955-021-01817-6)
Supplement: Supplementary file 9 — Additional file 9. Table 3. Sensitivity analysis to assess the inter-rater reliability for each one of second evaluators. [file 12955_2021_1817_MOESM9_ESM.docx]

## **Additional file 9. Table 3.** Sensitivity analysis to assess the inter-rater reliability for each one of second evaluators.

|  | Cohen´s kappa for second evaluator #1 | Cohen´s kappa for second evaluator #2 | higher kappa obtained by: |
| --- | --- | --- | --- |
| **Section 2. Patient wellbeing** | | | |
| 1. Is the patient experiencing unresolved physical symptoms  (including problems with breathlessness, pain, fatigue, nausea,  edema, insomnia, or cough)? | 0.38 | 0.46 | evaluator #2 |
| 2. Does the patient have problems with daily living activities? | 0.58 | 0.58 | equal |
| 3. Does the patient have psychological symptoms that are interfering with well-being or relationships? | 0.66 | 0.68 |  |
| 4. Does the patient have concerns about how to manage his/her medication and treatment regimens? | 1.00 | 0.48 | evaluator #1 |
| 5. Does the patient have concerns about spiritual or existential issues? | 0.88 | 0.88 | equal |
| 6. Does the patient have financial or legal concerns that are causing distress or require assistance? | 0.83 | 0.85 | evaluator #2 |
| 7. From the health delivery point of view, are there health beliefs, cultural, or social factors involving the patient or family that are making care more complex? | 0.00 | 0.20 | evaluator #2 |
| **Section 3. Ability of caregiver or family to care for patient** | | | |
| 1. Is the caregiver or family distressed about the patient’s physical symptoms? | 1.00 | 0.70 | evaluator #1 |
| 2. Is the caregiver or family having difficulty providing physical care? | 0.77 | 0.77 | equal |
| 3. Is the caregiver or family having difficulty coping? | 1.00 | 0.72 | evaluator #1 |
| 4. Is the caregiver having difficulty managing the patient’s medication and treatment regimens? | 1.00 | 1.00 | equal |
| 5. Does the caregiver or family have financial or legal concerns that are causing distress or require assistance? | 1.00 | 0.51 | evaluator #1 |
| 6. Is the family currently experiencing problems that are interfering with their functioning or interpersonal relationships or is there a history of such problems? | 0.86 | 0.44 | evaluator #1 |
| **Section 4. Caregiver wellbeing** | | | |
| 1. Is the caregiver or family experiencing physical, practical, spiritual, existential, or psychological problems that are interfering with their well-being or functioning? | 0.71 | 0.80 | evaluator #2 |
